# Development of Job Standards for Nurse‐Led Rapid Response Teams in the Republic of Korea

**DOI:** 10.1155/jonm/8558376

**Published:** 2026-09-15

**Authors:** Ju-Ry Lee, Ju Hee Lee, Yeon Soo Jang, Yeon Hee Kim, Eui Geum Oh

**Affiliations:** ^1^ Department of Nursing, Geoje University, Geoje 53325, Gyeongsangnam-do, South Korea; ^2^ Yonsei University Mo-Im Kim Nursing Research Institute, 50 Yonsei-ro Seodaemun-gu, Seoul 03722, South Korea; ^3^ University of Ulsan Department of Clinical Nursing, 88 Olympic-ro 43-gil, Seoul 05505, South Korea

**Keywords:** clinical competence, hospital rapid response team, nurse specialists, patient outcomes, standards

## Abstract

**Background:**

Nurse‐led rapid response teams (RRTs) significantly improve patient safety. However, the absence of specific job standards can precipitate role ambiguity and compromise care quality. Therefore, establishing tailored job standards that reflect the distinct healthcare context of the Republic of Korea is imperative to optimize RRT performance and clinical outcomes.

**Aim:**

To identify nurses′ activities in nurse‐led RRTs and to develop job standards that define the desired performance levels of RRT nurses and enable task evaluation within the Korean healthcare context.

**Methods:**

This study used a two‐step methodological approach, guided by a conceptual framework integrating Hamric’s advanced practice nursing model with nine Korean advanced practice nursing core competency domains: (1) identifying the nursing activities of nurse‐led RRTs through an integrative evidence review, benchmarking of domestic and international institutions, and in‐depth interviews with domestic nurses; and (2) developing draft standards and validating them through a two‐round Delphi survey and field verification of practical suitability.

**Results:**

A total of 210 potential activities were initially identified and refined into 55 nursing activities across nine core competency domains: advanced nursing practice, education, counseling, consultation, ethical decision‐making, research, collaboration, evidence‐based practice, and leadership. Draft job standards comprising 9 standards, 16 criteria, and 53 indicators were developed and validated through a two‐round Delphi survey with eight experts; after Round 1 revisions and two added indicators, all items met the retention criteria in Round 2 (I‐CVI ≥ 0.88, mean I‐CVI = 0.96, S‐CVI/Ave = 0.94). In field verification with 62 RRT nurses across 19 hospitals nationwide, all items showed practical suitability above 90%, and 9 standards, 16 criteria, and 55 indicators were ultimately confirmed. These standards integrate internationally accepted competency frameworks while reflecting the distinctive Korean context, such as EMR‐based screening and the absence of independent nurse prescriptive authority.

**Conclusion:**

This study identified nurse‐led RRT work activities and developed job standards for desired performance levels of RRT nurses. This provides a foundational framework for performance evaluation and continuous quality improvement. As a methodological study, it did not empirically test clinical outcomes; rather, the developed standards may support future outcome evaluations using metrics, such as cardiac arrest rates, unplanned ICU admissions, and failure‐to‐rescue, and may thereby provide a basis for advancing nursing care quality and professionalism in managing ward patient deterioration.

**Implications for Nursing Management:**

The job standards provide practical guidance for new and experienced RRT nurses and offer a foundation for defining job skills, structuring task instructions, workforce planning, and conducting performance evaluations across diverse hospital environments, while also informing qualification criteria. They may also serve as a useful framework to inform future empirical research evaluating the impact of nurse‐led RRTs on patient outcomes. To support consistent adoption, a phased implementation strategy involving nursing associations, professional bodies, and the Ministry of Health and Welfare is proposed.

## 1. Introduction

Rapid response teams (RRTs) were first conceptualized in the 1990s in Australia as a strategy to improve the early detection of clinical deterioration in ward patients and to provide timely interventions intended to reduce adverse events such as unplanned intensive care unit (ICU) admissions, in‐hospital mortality, and cardiac arrests [1]. Following the landmark publication by Lee et al. [2] and subsequent advocacy by the Institute for Healthcare Improvement’s 100,000 Lives Campaign, RRTs were rapidly adopted across North America, Europe, and the Asia‐Pacific region [1, 3]. The First Consensus Conference on Medical Emergency Teams (2005) established foundational principles for RRT implementation, emphasizing standardized team composition, activation criteria, and governance structures [1], and national patient safety initiatives have since endorsed RRTs as a core component of hospital safety infrastructure [3, 4].

Internationally, RRT models vary considerably in composition and operational design. A typical RRT is multidisciplinary, potentially including intensivists or other physicians, critical care nurses, respiratory therapists, pharmacists, and administrative staff, each contributing distinct competencies [1, 3]. The interdependence of these members is critical: Physicians provide medical decision‐making authority and procedural oversight; respiratory therapists manage ventilatory support; and nurses often serving as first responders and primary coordinators perform patient assessment, initiate interventions, liaise with ward staff, and coordinate the overall team response [1, 5, 6]. Recognizing this interdependence, several models have designated specific roles through standardized protocols and algorithms, thereby reducing role ambiguity and supporting efficient team performance [3, 7].

Among these configurations, nurse‐led models in which a nurse serves as team leader and primary decision‐maker during acute responses have shown promising effectiveness. Systematic reviews have reported associations between nurse‐led RRTs and reduced patient mortality [4], and individual studies have documented reductions in cardiac arrest rates and failure‐to‐rescue events, as well as improvements in staff satisfaction [6, 8–10]. In these models, the nurse leader receives the initial call from ward staff and provides immediate interventions, practicing advanced clinical nursing, educating and counseling patients and ward staff, and providing feedback on failure‐to‐rescue cases [2, 5, 8]. Even in nurse‐led models, however, the nurse leader’s effectiveness depends substantially on physician collaboration; internationally, such teams typically operate within frameworks that grant nurses expanded authority, including prescription rights and protocol‐driven interventions, while maintaining structured escalation pathways to physicians for complex decisions [9, 11].

In the Republic of Korea, RRTs were first introduced in 2008, when teams comprising physicians and clinical nurse specialists were deployed at tertiary hospitals in Seoul [12]. The system has since expanded substantially, and RRTs currently operate in 51 medical institutions nationwide [13]; at the time of this study (2018–2019), however, RRTs were operating in only 21 institutions nationwide. In 2019, the Ministry of Health and Welfare introduced a reimbursement scheme for RRTs to enhance inpatient safety and care quality [14]. The accompanying pilot project, implemented in April 2019, defined three types of RRTs based on personnel and equipment, with differential reimbursement; RRT nurses are a prerequisite for all three groups, two of which require only RRT nurses, while one requires both RRT nurses and a physicians [15]. This structure underscores the central role of nurses in the Korean RRT system while highlighting the variability in physician involvement across institutions.

The Korean RRT system differs from established international models in several respects. Whereas RRTs in the United States, the United Kingdom, and Australia have been established for over two decades, supported by national guidelines and accreditation standards, Korea’s system remains at a relatively early stage of standardization [1, 3, 12]. Most notably, Korean RRT nurses lack the independent prescriptive authority and protocol‐driven treatment autonomy commonly granted to their international counterparts [9, 11]. Instead, Korean RRTs employ electronic medical record (EMR)–based screening systems to detect deteriorating patients, enabling preemptive interventions such as proactive rounding, physical examination, and consultation with physicians [16]. Huh et al. [16] reported that this approach was associated with improved patient outcomes, though this evidence derives from a single institution. This regulatory difference reflects distinct institutional designs rather than a deficiency in either model; nonetheless, within the Korean model, the absence of standardized prescriptive authority and treatment protocols may delay interventions for deteriorating patients [17]. Table 1 presents an explicit comparison between Korea and the three countries with the longest established nurse‐led RRT systems across key dimensions: team composition, nurse prescriptive authority, activation mechanisms, and standardization of job standards. Beyond these structural and legal differences, organizational factors including established patterns of physician‐centered decision‐making and the relatively limited scope of national nursing association mandates shape how RRT nurses escalate concerns and exercise clinical judgment. Rather than reflecting fixed cultural traits, these are better understood as modifiable features of institutional workflow that can act as both enablers (e.g., team cohesion, adherence to protocol) and barriers (e.g., reluctance to challenge a ward physician’s plan).

**TABLE 1:** Comparison of nurse‐led RRT systems: Republic of Korea vs. international models.

Dimension	Republic of Korea	Australia	United Kingdom	United States
Year of introduction	2008 [13]	1990 (first MET, Liverpool Hospital) [1, 2]	Late 1990s–early 2000s [4]	2004–2005 (IHI 100,000 Lives Campaign) [1, 4]
National policy support	Ministry of Health and Welfare pilot project (2019); medical insurance fee established [15, 16]	National Safety and Quality Health Service Standards mandate recognition and response to clinical deterioration [4]	National Outreach Forum operational standards; NICE Clinical Guideline 50 (recommending graded response to deterioration) [4]	Joint Commission NPSG (2008); IHI campaigns [1, 4]
Typical team composition	RRT nurse required in all three reimbursement tiers; physician required in only one of the three [16]	Predominantly physician‐led MET; nurse‐led critical care outreach models exist in some hospitals [1, 4]	Critical care outreach team, typically nurse‐led; supported by on‐call intensivist [4]	Multidisciplinary: intensivist/physician, critical care nurse, respiratory therapist; nurse‐led models also exist [1, 5, 9]
Nurse’s legal/prescriptive authority	No independent prescription rights; no standardized treatment protocols for RRT nurses [18]	Nurse practitioners hold prescriptive authority under state/territory legislation	Nurse prescribing enabled under UK legislation (supplementary and independent prescribing) [11]	APRNs hold prescriptive authority in all 50 states, with full independent authority in approximately half [5, 6]
Activation/detection mechanism	Predominantly EMR‐based screening system (Early Warning Score + laboratory results); proactive rounding by RRT nurse [17]	Calling criteria based on single‐parameter or multiparameter track‐and‐trigger systems; mandatory calling policies [1, 4]	National Early Warning Score (NEWS/NEWS2) with graded escalation protocol [4]	Institution‐specific calling criteria; single‐parameter or aggregate‐weighted scoring systems [1, 4]
Key intervention approach	Preemptive interventions (proactive rounding, physical examination, bedside tests, ICU physician consultation) [17]	Direct bedside assessment and intervention by MET/RRT; protocol‐driven treatments [1]	Bedside assessment, stabilization, and facilitation of ICU transfer; education of ward staff [4]	Bedside assessment and stabilization; protocol‐driven interventions including medication administration [5, 6]
Nurse qualifications/certification	Varies by hospital (clinical nurse specialist, RRT nurses); no unified national standard [14, 16]	Critical care nursing experience required; some may hold nurse practitioner qualification (ACCCN Practice Standards; Nursing and Midwifery Board of Australia)	Critical care nursing experience; National Outreach Forum/CC3N competency framework [4]	Critical care registered nurse (CCRN); ACLS certification; APRN in some models [5]
Standardization of job standards	Not yet established	General competency standards for specialist critical care nurses exist, but no RRT‐specific job standards	Competency framework for critical care outreach practitioners; not RRT‐specific	ANA Scope and Standards of Practice for various specialties; no RRT‐specific national standard [18]

Job standards support work‐quality assessment by clarifying expected roles, objectives, and scope‐of‐practice criteria [18], whereas their absence can lead to overlapping roles, interprofessional conflict, and a potential decline in nursing quality [14, 19]. Therefore, taking into account the specific features of the Korean healthcare environment and the heterogeneity of RRT infrastructure across institutions [13, 15], there is a clear need to establish job standards for nurses in nurse‐led RRTs that are distinct from those for general nurses [17]. Such standards would enable RRT nurses to demonstrate their contributions more clearly and support appropriate institutional deployment. Hence, the purpose of this study was to develop job standards for nurses in nurse‐led RRTs so that they can demonstrate the desired levels of practice and professionalism [17].

## 2. Materials and Methods

### 2.1. Conceptual Framework

The conceptual framework of this study was constructed by integrating Hamric’s model of advanced practice nursing [20] with the core competency domains for Korean Advanced Practice Nurses (APNs), whose scope of practice is legally established under the Rules on the Qualification of APNs. The framework drew specifically on the core competencies of Korean APNs presented by the Korean Accreditation Board of Nursing Education (KABONE) [21], and the scope of practice and standards for APNs presented by the Korean Nurses Association (KNA) [22]. To preserve consistency with the nationally adopted competency nomenclature, the domain corresponding to Hamric’s “Direct Clinical Practice” is designated “Advanced Nursing Practice” in this framework, in alignment with the Korean APN competency standard of Advanced Direct Nursing Practice. Accordingly, the term “Advanced Nursing Practice” is used in this study to denote a specific subdomain within the framework rather than the overarching model, and this labeling choice is made explicit to avoid conceptual conflation between the model as a whole and one of its constituent domains.

**FIGURE 1:**
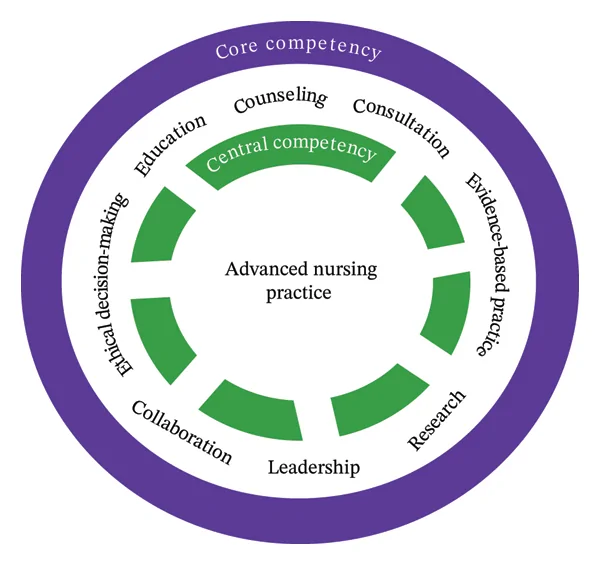
Conceptual framework of this study: Nurse‐led rapid response team competency.

On the basis of this integration, nine core competency domains were derived as shown in Figure 1: (I) Advanced Nursing Practice, (II) Education, (III) Counseling, (IV) Consultation, (V) Ethical Decision‐Making, (VI) Research, (VII) Collaboration, (VIII) Evidence‐Based Practice, and (IX) Leadership. These nine domains served as the structural categories under which the job activities and competency standards of nurse‐led RRTs were subsequently developed through the two‐step procedure described in Section 2.2.

Each of the nine competencies was defined at two levels: a conceptual definition, which specifies the theoretical meaning of the competency as articulated in Hamric’s APN model and KABONE core competency framework, and an operational definition, which specifies how each conceptual competency was contextualized for nurse‐led RRT practice in the present study. The operational definitions served as the a priori classification basis applied during the development of the job activity standards. The full set of conceptual and operational definitions, together with their mapping to Hamric’s APN model and the KABONE core competency framework, is provided in Supporting Table 1.

### 2.2. Study Design and Procedure

**FIGURE 2:**
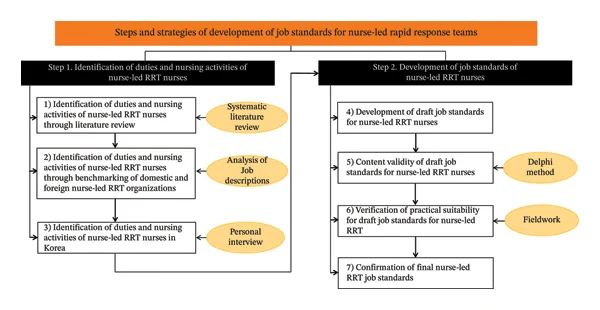
Steps and strategies in this study.

This study utilized a two‐step methodological procedure to develop job standards for nurse‐led RRTs, as shown in Figure 2. The procedure was structured to ensure that the derived standards were grounded in both the existing knowledge base and the practice realities of RRT nurses in Korea [17]. Step 1 established the preliminary pool of nursing activities through an integrative literature review, benchmarking of an institutional RRT program, and in‐depth interviews with domestic RRT nurses. Step 2 verified and refined the preliminary pool through a job analysis with currently practicing RRT nurses.

#### 2.2.1. Step 1. Identifying Activities in Nurse‐Led RRTs

The job scope and responsibilities of nurses in nurse‐led RRTs were identified through (1) an integrative evidence review, (2) integration of domestic and international RRT nurses’ job descriptions, and (3) in‐depth interviews with domestic RRT nurses. As multiple methods led to duplication, the researchers integrated and eliminated the duplicates, and the final activities were classified into nine core competencies, forming this study’s conceptual framework.

##### 2.2.1.1. Literature Review of Nurses’ Activities in Nurse‐Led RRTs

The researchers searched the following electronic databases to conduct an integrative evidence review to identify the roles and activities of nurse‐led RRTs as described in previous studies: PubMed, Cochrane Library, Cumulative Index to Nursing and Allied Health Literature (CINAHL), Google Scholar, KoreaMed, and Research Information Sharing Service (RISS). The National Institute for Health and Care Excellence and ProQuest Dissertations & Theses Global were searched for gray literature and hand‐searched by checking additional reference lists.

The review actively included gray literature, such as institutional job descriptions, national health agency policies, and professional guidelines from associations, such as the KNA, KABONE, and ANA, to mitigate publication bias and capture primary data that reflect real‐world clinical boundaries. The search terms for the PubMed database included controlled medical subject heading terms, and those for the EMBASE database included Emtree and free‐text terms. The search strategy was customized for each database using controlled vocabulary associated with the search terms (Supporting Table 2). Published languages were limited to English and Korean, and the years of publication were set from January 1, 1990 (when the first RRT was implemented) to December 28, 2018.

The reviewed literature encompassed quantitative and qualitative original studies, systematic reviews, descriptive studies, books, and editorials. In alignment with the study’s conceptual framework, the initial literature review focused strictly on publications explicitly detailing the competencies, roles, activities, and responsibilities of nurses within nurse‐led RRTs. Subsequently, a comprehensive supplementary search and manual screening process were integrated to encompass literature providing indirect references to RRTs or evaluating generalized rapid response system outcomes, thereby minimizing potential selection bias and enriching the overall findings. The complete literature selection process is illustrated in Supporting Figure 1.

##### 2.2.1.2. Benchmarking Against Domestic and International Nurse‐Led RRTs

We contacted nurse team leaders from 19 Korean hospitals operating nurse‐led RRTs as of November 2018 by email or telephone, explained the purpose of the study, and requested their nurses’ job‐description documents. These documents were collected via email from November 15 to 30, 2018. Similarly, to investigate nursing roles in overseas nurse‐led RRTs, we contacted nurses from 12 international hospitals in Australia (*n* = 4), the United States (*n* = 4), and the United Kingdom (*n* = 4) by email and, after explaining the purpose of the study, requested job‐description data. These countries were selected because they represent the longest established and most extensively documented nurse‐led RRT models internationally [1, 4–6]. These data were collected from December 1 to 15, 2018.

Integration of the domestic and international benchmarking data followed a structured, three‐stage process. First, two researchers independently extracted all nursing activities listed in the collected job‐description documents and organized them according to the nine core competency areas of this study’s conceptual framework. Second, the extracted items from domestic and international sources were placed side by side in a comparison matrix, and each item was coded as (a) common to both domestic and international job descriptions, (b) unique to domestic job descriptions, or (c) unique to international job descriptions. The resulting comparison matrix (Table 2) revealed critical differences between the two systems: Screening of deteriorating patients through an EMR‐based screening system is implemented domestically but is not commonly utilized abroad, whereas prescriptive authority is granted to RRT nurses abroad but is not legally permitted domestically. Third, items that were common or complementary were merged into unified duty statements, whereas items unique to either source were retained individually for further evaluation.

**TABLE 2:** Identify of nursing activities for nurse‐led rapid response teams.

Standard of competency	Nursing activities of nurse‐led RRTs		Source			
			Literature	Benchmarking of organization		In‐depth interviews
				Domestic	International	
I. Advanced nursing practice	1	Screening of deteriorating patients through screening system	○	○	X	○
	2	Assessment of current medical history, past history, and family history	○	○	○	○
	3	Identify and diagnostic tests and analyze results	○	○	○	○
	4	Data collection through interviews with patients and their families	○	○	○	○
	5	Data collection through interviews with ward medical staff	○	○	○	○
	6	Proactive rounding	○	○	○	○
	7	Perform physical examination	○	○	○	○
	8	Continuous monitoring of physiological status changes such as blood pressure, heart rate, respiration rate, and oxygen saturation	○	○	○	○
	9	Nursing diagnosis by integrating and analyzing the assessed data establish	○	○	○	○
	10	Differential diagnosis	○	○	○	○
	11	Establishing a nursing plan in collaboration with patients, families, and medical staff	○	○	○	○
	12	Individualized, evidence‐based treatment and nursing intervention planning that takes into account the needs of patients and families	○	○	○	○
	13	Establishing an appropriate nursing plan according to the condition of the deteriorating ward patients	○	○	○	○
	14	Management or intervention according to the situation of deteriorating ward patients	○	○	○	○
	15	Management of patient with emergency airway problem	○	○	○	○
	16	Management of patient with cardiac arrest	○	○	○	○
	17	Management of patient with sepsis/septic shock	○	○	○	○
	18	Management of patient with shock	○	○	○	○
	19	Management of patient with altered mentality	○	○	○	○
	20	Management of patients who discharge from the intensive care unit	○	○	○	○
	21	Perform diagnostic tests	○	○	○	○
	22	Treatment, procedure performance, and management	○	○	○	○
	23	Prescription drugs and laboratory tests	○	X	○	X
	24	Evaluation of the appropriateness of treatment and nursing intervention	○	○	○	○
	25	Evaluation of outcomes for treatment and nursing intervention	○	○	○	○
	26	Revise and supplement plans and interventions according to evaluation results	○	○	○	○

II. Education	27	Education for patients and their families	○	○	○	○
	28	Education for nurses	○	○	○	○
	29	Development and evaluation of educational program	○	○	○	○

III. Counseling	30	Providing necessary information and counseling to patients, families, and nurses	○	○	○	○

IV. Consultation	31	Providing advice to ward doctors	○	○	○	○
	32	Providing advice to ward nurses	○	○	○	○
	33	Consultation with experts regarding patient treatment	○	○	○	○

V. Ethical decision‐making	34	Providing patients and their families with factual, evidence‐based information about the patient’s condition, examinations, procedures, and treatment plans	○	○	○	○

VI. Research	35	Conducting leading nursing research	○	○	○	○
	36	Conducting collaborative research	○	○	○	○
	37	Presentation of research results through conference presentations and publications	○	○	○	○

VII. Collaboration	38	Sharing information about patient status, treatment plan, and goals with ward medical staff	○	○	○	○
	39	Establish a work collaboration system with ward medical staff	○	○	○	○

VIII. Evidence‐based practice	40	Choosing the best research results	○	○	○	○
	41	Perform evidence‐based practice	○	○	○	○
	42	Evidence‐based performance evaluation after practice	○	○	○	○

IX. Leadership	43	Perform quality improvement activities	○	○	○	○
	44	Data collection on rapid response teams’ activities and outcomes	○	○	○	○
	45	Report on the activities of the rapid response team through regular meetings	○	○	○	○
	46	Review the patient safety cases and identify the cause of the problems found and come up with an improvement plan	○	○	○	○
	47	Management of evaluation indicators for rapid response team activities	○	○	○	○
	48	Management of clinical quality indicators (cardiopulmonary resuscitation indicators, etc.).	X	○	X	○
	49	Providing feedback to ward medical staff and conducting on‐site training on cases of failure‐to‐rescue	○	○	○	○
	50	Participate in the development and revision of clinical treatment (practical) guidelines or protocols (algorithms)	○	○	○	○
	51	Development, application, and effectiveness evaluation of an EMR‐based screening system for early detection of high‐risk ward patients with deterioration	X	○	X	○
	52	Participation in policy‐making activities to improve patient safety	○	○	○	○
	53	Continuous participation in practical competency education and professional group activities for professional development of nurses in the rapid response team	○	○	○	○
	54	A nurse’s mentor role for professional nursing practice for deteriorating ward patients	○	○	○	○
	55	Adjustment of necessary resources in case of deteriorated patients in the ward	○	○	○	○
	56	Management of supplies, medicines, medical devices, and documents	○	○	○	○

Total			54	48	53	55

*Note:* ○ = activity identified in this source; X = activity not identified in this source. Lit. = literature; CPR = cardiopulmonary resuscitation.

Abbreviations: EMR, electronic medical record; ICU, intensive care unit; RRT, rapid response team.

No differential weighting was applied to domestic versus international sources at this stage; that is, all benchmarking data regardless of country of origin were treated equally during the extraction and categorization process. The rationale for equal treatment was that the purpose of this step was to generate as comprehensive an inventory of potential activities as possible, rather than to prioritize one healthcare context over another.

Differential contextualization was instead applied at a later stage: During the development of draft job standards (Step 2), items that were prescription‐related, applicable only to international nurse‐led RRTs, or not legally sanctioned in the Korean healthcare environment were excluded (see Section 3.1). This two‐stage approach equal‐weight extraction followed by context‐specific filtering ensured that the final standards reflected international best practices while remaining feasible within Korean legal and institutional constraints.

##### 2.2.1.3. Identification of Nurses’ Activities in Nurse‐Led RRTs in Korea

Since there is no literature on nurses′ roles and activities in nurse‐led RRTs in Korea, the researchers conducted the job analysis using the personal interview method, visiting and interviewing highly skilled personnel regarding their respective tasks. The intentional extraction method was used to select individuals with the expertise required by the researchers.

Participants in the personal interviews were nurses and team leaders with more than three years of experience in nurse‐led RRTs. After the researchers had explained the study’s purpose, the participants confirmed their intention to participate and gave consent. The research data were collected through face‐to‐face interviews from January 4 to 30, 2019. To build rapport with the study participants, at the beginning of the interview, the researchers shared how they had developed an interest in the subject.

Data were analyzed using content analysis, a method that employs inductive analysis to identify topics or repeating patterns and interprets text content subjectively through a systematic classification process [23]. When literature or existing theories are lacking, this method is effective for classifying large quantities of data and eliciting categories of similar meanings [23, 24]. Given the lack of studies on nurses’ activities and roles in nurse‐led RRTs in Korea, content analysis was suitable for this study.

The qualitative content analysis procedure was as follows. First, nine areas of advanced nursing practice were established based on the core competencies of RRT nurses: advanced nursing practice, education, counseling, consultation, collaboration, research, evidence‐based practice, ethical decision‐making, and leadership. Second, coding was based on repeatedly reviewing the verbal content of participants′ responses to open questions by category, analyzing only the activities and roles of the nurses in the responses. Third, similar content was combined into meaningful expressions, and common themes were identified. Fourth, participants’ responses were used to determine the frequency of common topics.

#### 2.2.2. Step 2. Developing Nurses’ Job Standards in Nurse‐Led RRTs

##### 2.2.2.1. Draft Job Standards

Based on the RRT nurses’ activities identified in Step 1, draft job standards comprising standards, criteria, and indicators were developed on the basis of the nine core competencies of nurses in nurse‐led RRTs. These activities had been identified through a comprehensive analysis encompassing the integrative evidence review, domestic benchmarking, international benchmarking, and in‐depth interviews with domestic RRT nurses (Table 2). In total, 56 nursing activities were initially identified across the nine competency domains, with broad consensus observed across all four analytical sources for the majority of items; following context‐specific filtering to exclude activities not sanctioned within the Korean legal and institutional scope of practice, 55 activities were finalized. A narrative summary of the cross‐source agreement by domain is provided in Section 3.1, whereas the detailed activity‐level matrix is retained in Table 2, which provides an audit trail linking each final indicator to its evidence sources.

##### 2.2.2.2. Content Validity of the Draft Job Standards

The content validity of the draft job standards was verified through a two‐round Delphi survey conducted with an expert panel. Each round used a structured questionnaire containing the full set of draft standards (with their conceptual definitions), criteria, and indicators. Experts rated the relevance of each item on a four‐point Likert scale (4 = very relevant, 3 = relevant, 2 = somewhat relevant, 1 = not relevant) and were further asked to provide written qualitative comments on the wording, classification, and completeness of each item. For items rated 1 or 2, experts were required to suggest alternative wording or propose revisions.

Content validity was evaluated using both the item‐level content validity index (I‐CVI) and the modified kappa coefficient (*κ* ^∗^) to control for chance agreement [25–27]. At the scale level, the scale‐level CVI averaging calculation (S‐CVI/Ave) was computed as the mean of all I‐CVI values [25]. The prespecified criteria for retaining an item were I‐CVI ≥ 0.80 and *κ* ^∗^ > 0.74, the latter representing the threshold for “excellent” content validity [25–27]; the criterion for acceptable scale‐level content validity was S‐CVI/Ave ≥ 0.90.

Items meeting both criteria in Round 1 were retained without modification. Items with I‐CVI < 0.80 or *κ* ^∗^ ≤ 0.74 in Round 1 were revised based on the experts′ qualitative comments and re‐rated in Round 2, together with any additional items proposed by the panel. Inter‐round expert convergence was monitored by computing the mean absolute change in I‐CVI between Round 1 and Round 2. Data were collected either via email or through in‐person distribution by the researcher. The data collection for Round 1 was conducted from April 1 to April 20, 2019, and for Round 2 from April 20 to April 30, 2019.

##### 2.2.2.3. Verification of Draft Job Standards’ Practical Suitability

We verified the practical suitability of the draft job standards whose criteria and indicators had demonstrated content validity. At the time of this study (2018–2019), 21 institutions nationwide operated nurse‐led RRTs; of these, two whose RRTs were not exclusively nurse‐staffed were excluded, and the remaining 19 institutions participated in this verification. Sixty‐two RRT nurses or clinical nurse specialists working in RRTs at 19 of 21 nationwide hospitals participated in this verification; two hospitals whose RRTs were not exclusively nurse‐staffed were excluded. The nurse specialists assessed the practical suitability of each criterion using a four‐point Likert scale (4 = very suitable, 3 = suitable, 2 = not suitable, 1 = not suitable at all). Questionnaires were distributed and retrieved via email with the participants′ consent, yielding 62 completed responses (100% response rate) between April 20 and May 10, 2019. To broaden representation beyond academically affiliated experts, the 62 RRT nurses were recruited from institutions of varied size and geographic location (tertiary hospitals in both capital and noncapital regions, as well as general hospitals) and with varied levels of RRT clinical experience (ranging from less than 1 year to more than 10 years), so that the perspectives of less‐experienced nurses and those in nonacademic settings were also represented in the field‐validation step.

##### 2.2.2.4. Confirmation of the Final Job Standards

The job standards were finalized on the basis of the verification of the practical‐suitability results.

### 2.3. Ethical Considerations

Ethical approval was granted by the Institutional Review Board of Asan Medical Center (Protocol Code: S2018‐1810‐0001) on June 23, 2018, and the researchers contacted the nursing divisions at each healthcare facility before data collection to explain the study’s purpose and methods and request cooperation. Participants received a document explaining the purpose and methods of the study and guaranteeing anonymity, and they were asked to sign an informed consent form. The researchers informed participants that they were free to withdraw from the study at any time. To ensure anonymity, a secure envelope was provided for participants to return the questionnaires, and no one other than the study’s researchers was allowed to read the completed questionnaires. The research database was stored on a computer with restricted access to prevent exposure to the outside world; when creating documents or managing data, encryption was used to ensure confidentiality.

## 3. Results

### 3.1. Results Identifying Nurses’ Activities in Nurse‐Led RRTs

A total of 210 potential activities for nurses in nurse‐led RRTs were initially identified: 54 from the expanded literature review, 101 from domestic and international benchmarking, and 55 from in‐depth interviews with domestic RRT nurses. After removing 154 duplicate items, the remaining 56 distinct activities demonstrated robust cross‐source validity: 54 items (96.4%) were supported by the literature, 48 (85.7%) by international benchmarking, 53 (94.6%) by domestic benchmarking, and 55 (98.2%) by the in‐depth interviews. Following a context‐specific filtering stage to exclude tasks involving independent prescriptive authority or those legally restricted within the Korean healthcare framework, 55 activities were ultimately finalized across the nine core competency domains. Table 2 presents the complete activity‐level matrix as an audit trail, and the specific consensus patterns observed across the sources are detailed below.

#### 3.1.1. Advanced Nursing Practice

Advanced nursing practice in this study refers to the case‐level clinical performance carried out at the bedside of an individual deteriorating ward patient. Its scope is bounded by a single patient encounter and is operationalized through activities of clinical reasoning and direct care: screening, history‐taking, physical examination, continuous physiological monitoring, differential diagnosis, planning, intervention, and evaluation of that patient’s outcomes. Throughout, these activities are understood to be performed within the Korean legal and institutional scope of nursing practice, that is, under physician order, standing protocol, or collaborative RRT arrangements, rather than through independent nurse prescribing. The unit of analysis is the patient, and the temporal horizon is the duration of the acute deterioration episode. The largest competency domain comprised 26 activities related to advanced nursing practice. These included screening of deteriorating patients through a screening system; assessment of present, past, and family medical history; collecting and interpreting diagnostic test results ordered under physician direction or institutional protocol; data collection through interviews with patients, families, and ward medical staff; proactive rounding; physical examination; and continuous monitoring of physiological parameters such as blood pressure, heart rate, respiratory rate, and oxygen saturation. Higher order clinical reasoning activities included formulating nursing diagnoses through integration and analysis of assessment data; performing differential diagnosis; establishing nursing plans in collaboration with patients, families, and medical staff; and developing individualized, evidence‐based treatment and nursing intervention plans tailored to the condition of deteriorating ward patients.

Within this domain, nurse‐led RRTs were also responsible for managing or intervening in a range of acute clinical situations, specifically patients with emergency airway problems, cardiac arrest, sepsis or septic shock, shock, altered mentation, and patients discharged from the ICU, as well as performing diagnostic tests and treatments and procedures under physician order, established institutional protocol, or collaborative arrangement. Evaluation activities included assessing the appropriateness and outcomes of treatment and nursing interventions, and revising nursing plans accordingly. Notably, the activity of prescribing medications and laboratory tests (Item 23) was supported by the literature and international institutional benchmarking but was not identified in the domestic institutional benchmarking and in‐depth interviews with RRT nurses, reflecting the regulatory limits on independent prescriptive authority for nurses in the Korean context.

#### 3.1.2. Education

Three educational activities (Items 27–29) were identified: providing education to patients and families, delivering education to ward nurses, and developing and evaluating educational programs. All three were confirmed across all sources.

#### 3.1.3. Counseling

One counseling activity (Item 30) was identified: providing necessary information and counseling to patients, families, and ward nurses. This was consistently supported by all four analytical sources.

#### 3.1.4. Consultation

Three consultation activities (Items 31–33) were identified: providing advice to ward physicians, providing advice to ward nurses, and consulting with specialists regarding patient treatment plans. All three were confirmed across all four sources.

#### 3.1.5. Ethical Decision‐Making

One activity was identified (Item 34): providing patients and families with factual, evidence‐based information regarding the patient’s condition, examinations, procedures, and treatment plans. This was endorsed by all sources.

#### 3.1.6. Research

Three research activities (Items 35–37) were identified: conducting independent nursing research, participating in collaborative research, and disseminating findings through conference presentations and publications. All three were supported by all four sources.

#### 3.1.7. Collaboration

Two collaborative activities (Items 38–39) were identified: sharing information about patient status, treatment plans, and goals with ward medical staff, and establishing systematic work collaboration with ward medical staff. Both activities were confirmed across all four analytical sources.

#### 3.1.8. Evidence‐Based Practice

Three activities (Items 40–42) were identified: selecting the best available research evidence, implementing evidence‐based practices, and evaluating the outcomes of evidence‐based performance. All three were consistently endorsed across all sources. Although evidence‐based practice and advanced nursing practice both involve clinical judgment, they remain conceptually distinct (see Introduction): the former concerns how research evidence is appraised and integrated, whereas the latter concerns the direct clinical actions performed at the bedside.

#### 3.1.9. Leadership

The leadership domain comprised 14 activities (Items 43–56), the broadest scope of any single domain. These included quality improvement activities, data collection on RRT activities and outcomes, regular reporting through team meetings, patient safety case review and improvement planning, management of evaluation indicators, and on‐site training and feedback for ward staff on failure‐to‐rescue cases. RRT nurses were also responsible for participating in the development and revision of clinical guidelines and protocols and in patient safety policy‐making. Additional leadership responsibilities included professional development through continuing education, serving as mentors for ward nurses, coordinating resources for deteriorating patients, and managing supplies, medications, medical devices, and related documentation. Notably, two activity managements of clinical quality indicators such as cardiopulmonary–resuscitation metrics (Item 48) and development and application of EMR‐based screening systems for the early detection of high‐risk patients (Item 51) were not identified through the literature review or international institutional benchmarking, but were confirmed by both domestic institutional benchmarking and in‐depth interviews with domestic RRT nurses.

### 3.2. Job Standards of Nurse‐Led RRTs

#### 3.2.1. Development of the Draft Job Standards for Nurse‐Led RRTs

The draft job standards comprised 9 standards, 16 criteria, and 53 indicators. The 9 draft standards were developed based on the core competencies for nurse‐led RRTs; the 16 draft criteria represented nursing activities that could be used to evaluate the achievement of the draft standards; and the 53 draft indicators could be used to evaluate the achievement of the draft criteria.

#### 3.2.2. Content Validity of Draft Job Standards

The expert panel comprised eight specialists with established expertise in RRT practice and critical care, including three doctorate‐prepared nursing professors and five master’s‐prepared clinical nurse specialists. The panel was further composed by professional role as follows: four RRT specialist nurses, each holding a master’s degree, with approximately 15 years of total clinical experience and at least 10 years of RRT‐specific experience; the president of the Korean Rapid Response System Research Association (a physician); the director of the same association (a specialist nurse); and two full‐time physician professors involved in RRT operations at a general hospital. This composition was designed to ensure complementary expertise across nursing practice, medical practice, and the professional governance of RRT systems in Korea.

##### 3.2.2.1. Round 1

In Round 1, all 9 draft standards, 16 draft criteria, and 53 draft indicators were evaluated by the expert panel. I‐CVI values ranged from 0.75 to 1.00, with corresponding modified kappa (*κ* ^∗^) values from 0.72 to 1.00. Eight indicators received an I‐CVI of 0.75 (i.e., agreement from 6 of 8 experts; *κ* ^∗^ = 0.72, “good”), falling below the prespecified retention criteria of I‐CVI ≥ 0.80 and *κ* ^∗^ > 0.74 (“excellent”); these items were revised based on the experts’ qualitative comments. In addition, two new indicators proposed by the panel one concerning ventilator management within the advanced nursing practice domain and one concerning participation in healthcare‐policy development within the leadership domain were added to the draft. At the scale level, the mean I‐CVI was 0.91, the mean *κ* ^∗^ was 0.90 (“excellent”), and the S‐CVI/Ave was 0.91.

##### 3.2.2.2. Round 2

In Round 2, the revised draft comprising 9 standards, 16 criteria, and 55 indicators (the 53 original indicators with 0 deletions and 2 additions) was re‐rated by the same expert panel. All items achieved I‐CVI ≥ 0.88 and *κ* ^∗^ ≥ 0.88 (“excellent”), with 47 of 55 indicators (85.5%) achieving an I‐CVI of 1.00 (*κ* ^∗^ = 1.00). The mean I‐CVI was 0.96, the mean *κ* ^∗^ was 0.96 (“excellent”), and the S‐CVI/Ave was 0.94. The mean absolute change in I‐CVI between Round 1 and Round 2 was 0.07, indicating substantial convergence of expert agreement following the Round 1 revisions. As all items satisfied the prespecified content validity criteria, no further revisions were required, and the content‐validated draft comprising 9 standards, 16 criteria, and 55 indicators proceeded to the subsequent practical‐suitability verification step.

#### 3.2.3. Practical Suitability Test for the Draft Job Standards

This study used fieldwork to test the content‐validated draft job standards. A total of 62 RRT nurses from across the country participated. The participants’ average age was 33.7 years; the sample comprised 13 men (21.0%) and 49 women (79.0%). Regarding education level, 29 had bachelor’s degrees (46.8%), 16 had master’s degrees (25.8%), and 17 had completed some graduate courses (27.4%). Average total clinical experience at medical institutions was 10.3 years, with 35.5% of participants having 10–15 years of experience. Average RRT clinical experience was 3.2 years, with 30.6% having less than 1 year. The analysis showed that the practical suitability of all items in the drafts was greater than 90%. Specific results are presented below.

#### 3.2.4. Results for Draft Standards

The conformity analysis showed that the average practical suitability of the nine standards was 99.6%, ranging from a minimum of 98.4% to a maximum of 100%. Standards with 100% practical suitability were as follows: advanced nursing practice, education, counseling, collaboration, ethical decision‐making, evidence‐based practice, and leadership. The standards with the highest average scores for practical suitability were advanced nursing practice (3.9 ± 0.3) and collaboration (3.9 ± 0.3), followed by education (3.8 ± 0.4), ethical decision‐making (3.8 ± 0.4), and leadership (3.8 ± 0.4). Counseling (3.6 ± 0.5) and research (3.6 ± 0.5) had the lowest average practical‐suitability score, at 98.4% practical suitability.

### 3.3. Results for the Draft Criteria

Analysis of the practical suitability of the 16 draft criteria yielded an average practical suitability of 99.0%, ranging from 91.9% to 100%. Of the 16 draft criteria, 10 had the highest practical suitability, and the highest average conformity scores were observed for Criterion 1, “Assessing the deteriorating ward patients” (3.9 ± 0.3), and Criterion 10, “Building a collaborative relationship with the medical team involved in treating and nursing the deteriorating ward patients” (3.9 ± 0.3).

### 3.4. Results for the Draft Indicators

The analysis showed that the average practical suitability of the 55 indicators was 98.7%, ranging from 91.9% to 100%. A total of 38 draft indicators demonstrated 100% practicality, and the highest average score was 3.9 ± 0.3 for the following draft indicators: Indicator 1, “Comprehensive data collection is carried out for patients at high risk of deterioration in the ward”; Indicator 9, “Patients with dyspnea provide appropriate intervention and nursing”; Indicator 10, “Operating and administering oxygen therapy”; Indicator 12, “Conducting emergency airway management”; Indicator 15, “Care of shock patients”; Indicator 36, “Sharing information on patients’ condition and treatment plans and goals with the ward medical team”; Indicator 37, “Cooperating with the RRT doctors by establishing a task‐collaboration system”; and Indicator 38, “Working with the hospital staff.” Among the 17 items that did not score 100%, the indicator with the lowest average practical suitability was Indicator 27, “Evaluation of educational effectiveness” (3.3 ± 0.7, 91.9%).

### 3.5. Finalization of Job Standards for Nurse‐Led RRTs

Verification of the practical suitability of the draft job standards revealed that the conformity of practice was 90% or greater for all questions on the 9 draft standards, 16 criteria, and 55 indicators. The nurses’ draft job standards were deemed suitable for practice and finalized (Table 3).

**TABLE 3:** Final developed job standards for nurse‐led rapid response teams in Korea.

Standards	Criteria	Indicator	
1. Advanced nursing practice Nurse‐led RRTs provide advanced clinical practice based on expertise and skills for deteriorating ward patients	1. Assess deteriorating ward patients.	1.	Perform comprehensive data collection on high‐risk patients with deterioration in the general ward.
		2.	Screening for deteriorating ward patients.
	2. Integrate and analyze the assessed data to make nursing diagnosis.	3.	Differential diagnosis of similar diseases related to clinical symptoms, under collaborative arrangements
		4.	Identifying nursing diagnoses for actual and potential nursing problems.
		5.	Participate in the establishment of a medical diagnosis through consultation with doctor.
	3. Develop a nursing plan needed to achieve nursing goals.	6.	Set nursing and treatment goals based on nursing diagnosis.
		7.	Plan appropriate treatments and interventions based on priorities for deteriorating ward patients.
		8.	Make nursing plans for the expected results.
	4. Perform advanced nursing practice in accordance with the established nursing plan.	9.	Provide appropriate interventions to patients with dyspnea.
		10.	Apply and manage oxygen therapy.
		11.	Apply and manage artificial ventilator
		12.	Care of patient with airway emergency.
		13.	Care of cardiac arrest patients.
		14.	Care of sepsis patients.
		15.	Care of shock patients.
		16.	Care of patients with altered mentality.
		17.	Care of patients with discharge from ICU.
		18.	Administer prescribed drug therapy under physician orders, established protocols, or collaborative arrangements
		19.	Perform diagnostic tests under physician orders, established protocols, or collaborative arrangements
		20.	Perform and care for treatments and procedures.
	5. Evaluate treatment and nursing intervention.	21.	Evaluate the appropriateness of treatment and nursing interventions.
		22.	Evaluate progress to planned goals.
		23.	Evaluate result of treatment and nursing intervention.
		24.	Revise and supplement plans and nursing interventions according to evaluated results.

2. Education Nurse‐led RRTs educate the clients based on their expertise.	6. Educate the patients or families.	25.	Assess the educational needs of the patients and families.
		26.	Educate the patients and families.
		27.	Evaluate the effectiveness of education.
	7. Educate the nurses.	28.	Assess the educational needs of ward nurses.
		29.	Educate the ward nurses.
		30.	Develop educational programs.
		31.	Evaluate the effectiveness of education.

3. Counseling Nurse‐led RRTs provide counseling to subjects to identify clients’ needs and resolve problems.	8. Provide counseling to the clients.	32.	Provide counseling to patients and families.

4. Consultation Nurse‐led RRTs help to solve problems for patients, staff, or systems using their expertise.	9. Provide advice and consult other experts if necessary.	33.	Respond to medical staff for deteriorating ward patients.
		34.	Respond to other departments or other medical institutions for the rapid response system.
		35.	Consult with experts in the field (e.g., intensive care unit doctors, etc.) as needed to ensure that the deteriorating ward patients receive appropriate treatment.

5. Collaboration Nurse‐led RRTs collaborate with people who provide health care to achieve desirable outcomes when treating deteriorating ward patients	10. Establish and maintain collaborative relationships with medical staff involved in the treatment and intervention of ward patients with deterioration.	36.	Share information about ward medical staff and patient conditions, treatment plans, and goals.
		37.	Establish a collaborative system with the ward medical staff to carry out practical work for the deteriorating ward patients.
		38.	Collaborate with the RRT physician.

6. Ethical decision‐making Nurse‐led RRTs identify ethical issues about patient care and support patient rights and safety	11. Advocate for the rights and safety of patients.	39.	Provide patient and family factual information about the patient’s condition changes, results of laboratory, procedures, and treatment plans.
		40.	Respect patients and families’ involvement.
		41.	Respect the right to choose and refuse treatment and care for patients and their families.

7. Research Nurse‐led RRTs seek professional qualities by participating in research activities that can continue to develop expertise.	12. Conduct research activities.	42.	Design and conduct a clinically focused research.
		43.	Present the results of research.

8. Evidence‐based practice Nurse‐led RRTs incorporate best‐in‐class research and clinical experience and apply evidence in consideration of patient preferences when making decisions about patient care and nursing interventions.	13. Perform evidence‐based practice.	44.	Identify the best results of research.
		45.	Choose intervention based on the best evidence that takes into account clients’ goals and priorities.
		46.	Apply the chosen intervention to practice.
		47.	Apply the chosen intervention to practice and evaluate the results.

9. Leadership Nurse‐led RRTs demonstrate clinical leadership to improve the quality of care and nursing interventions and advanced nursing practice provided to ward patients with deterioration.	14. Improve the quality of treatment and nursing care for deteriorating ward patients.	48.	Review patient safety cases and identify the cause of the problem to derive improvements.
		49.	Manage RRT activity evaluation indicators.
		50.	Participate in the development and modification of clinical practice guidelines or protocols (algorithms) related to the treatment and nursing intervention of ward patients with deterioration.
	15. Develop professionalism and work to improve it.	51.	Improve professionalism through self‐development
		52.	Become a nurse’s role model as a professional.
		53.	Participate in establishing healthcare policies related to deteriorating ward patients and the rapid response system.
	16. Coordinate and manage the resources and skills necessary to effectively respond to crisis conditions associated with deteriorating ward patients.	54.	Coordination and management of the necessary human and material resources in case of deteriorating ward patients.
		55.	Manage drugs, medical devices, and documentation.

## 4. Discussion

This methodological study aimed to develop job standards for nurse‐led RRT practice in Korea. Through the integration of an integrative evidence review, institutional benchmarking, and a job analysis with currently practicing RRT nurses, an initial pool of activities was refined into 55 nursing activities across nine competency domains: advanced nursing practice, education, counseling, consultation, ethical decision‐making, research, collaboration, evidence‐based practice, and leadership. The content‐validated and field‐tested final standards comprised 9 standards, 16 criteria, and 55 indicators. The study revealed that nurses in domestic nurse‐led RRTs exhibit distinct practice patterns compared to their international counterparts in two key aspects: the utilization of EMRs for screening deteriorating ward patients and the absence of prescriptive authority. These two features are examined in turn below, as each carries distinct implications for how the proposed standards operate within the Korean context.

The first distinction concerns the mechanism by which deterioration is recognized and the RRT activated. To improve the outcomes of deteriorating ward patients, hospital staff must recognize deterioration‐related symptoms at an early stage and activate the RRT [3]. In several countries, RRT activation criteria are defined by the clinical characteristics of deteriorating patients, and afferent‐limb systems have been established that mandate RRT activation once predefined thresholds are met [3]. However, the mandatory‐activation model is not without tension: Some ward physicians have expressed concern that automatic calls may diminish their opportunity to provide primary management of deteriorating patients themselves [28, 29]. In addition, several studies have reported that, in the absence of explicit direction from the ward physician, ward nurses may be reluctant to initiate a mandatory RRT call when a patient’s condition deteriorates [30–33], a reluctance often linked to interprofessional communication barriers and concerns about conflict. Taken together, these findings indicate that the effectiveness of mandatory‐activation systems depends not only on well‐defined criteria but also on the organizational and interprofessional context in which they operate.

Partly in response to these interprofessional and workflow challenges, Korean hospitals operating RRTs have widely adopted EMR‐based screening systems rather than relying solely on conventional manual triggers. Notably, this shift reflects not only clinical preference but also health‐policy incentives: under the ongoing rapid response.

System pilot project, operating an EMR‐based screening system, is a required criterion for hospitals to qualify for National Health Insurance (NHI) reimbursement [14, 15]. As a result, EMR‐based screening has become the de facto standard for RRT operation in Korea. This screening mechanism facilitates the early detection of physiological deterioration by integrating both abnormal vital signs and laboratory data. Upon identifying at‐risk patients through this automated surveillance, RRT nurses initiate preemptive interventions, including proactive rounding, targeted physical examinations, bedside diagnostics, and, when necessary, consultation with intensive care physicians [16]. In support of this approach, Huh et al. [16] reported that proactive screening and preemptive management by clinical nurse specialists were associated with improved patient outcomes, although, as a single‐institution study, these findings warrant cautious generalization. Taken together, EMR‐based continuous screening driven by both clinical rationale and the reimbursement requirements of the pilot project constitutes a defining nursing activity that distinguishes nurse‐led RRTs in Korea from the manual‐activation models used in many other countries.

The second distinction concerns the scope of intervention available to RRT nurses once deterioration is identified. In several countries, nurses in nurse‐led RRTs may hold prescriptive authority as APNs, which can enable more timely interventions for deteriorating patients [3, 5, 11]. In these settings, a number of international institutions with nurse‐led RRTs have expanded nursing scopes of practice by implementing protocol‐driven prescriptive authority [3, 5, 11]. Specifically, RRT nurses in these settings are equipped with treatment algorithms for managing acute conditions such as chest pain, seizures, shock, and altered mental status; these protocols provide a legal basis for nurses to issue targeted prescriptions under specified clinical circumstances [5, 11]. By contrast, nurse‐led RRTs in Korea operate primarily by facilitating the early recognition of clinical deterioration through EMR‐based screening systems [16]. These divergent models should be understood not as a simple hierarchy of superiority but as distinct institutional designs, each reflecting the particular regulatory environment, legal scope of nursing practice, and clinical infrastructure of its healthcare system.

Prescription‐enabled intervention in the former and technology‐supported early detection in the latter. At present, domestic RRT nurses hold neither independent prescriptive authority nor standardized treatment protocols tailored to deteriorating patients, which, within this model, may contribute to delays in time‐sensitive interventions. Therefore, to support patient outcomes and enable RRT nurses to carry out their clinical responsibilities more fully within the team, the expansion of legal and institutional frameworks merits consideration. Such frameworks could define the conditions under which RRT nurses may initiate specific prescriptions, apply clinical protocols, and deliver essential treatments under strictly defined clinical circumstances, provided that such expansion is accompanied by appropriate education, credentialing, and safeguards for patient safety.

Although this study’s primary product is a set of job standards rather than a patient‐outcome trial, the patient‐centered rationale underlying the standards is central to their justification. Internationally, nurse‐led RRTs have been associated with reductions in in‐hospital mortality [4], lower cardiac arrest rates outside the ICU [6, 10], and reduced unplanned ICU admissions and failure‐to‐rescue [8, 9]. By codifying the standards, criteria, and indicators that operationalize these interventions, the standards developed here may provide a mechanism for translating internationally reported outcome benefits into more reproducible clinical practice in Korean hospitals.

### 4.1. Cultural and Systemic Influences on Implementation

Beyond legal and infrastructural differences, organizational and systemic factors substantially shape how the proposed job standards can be implemented across healthcare contexts. We discuss these factors at three levels, the organizational, the regulatory, and the cross‐national, and identify the corresponding implementation barriers and facilitators for each.

At the organizational level, established patterns of physician‐led clinical decision‐making in Korean tertiary‐care hospitals can influence how readily RRT nurses act on their assessments. Even when clinically expert, RRT nurses may at times defer to a ward physician’s initial plan when patient deterioration is evident, which can attenuate the effectiveness of indicators in the collaboration and consultation domains. Rather than attributing this pattern to broad cultural traditions, it is more usefully understood as a modifiable feature of institutional workflow: structured, low‐threshold communication tools such as SBAR‐based escalation scripts and preauthorized activation criteria can depersonalize escalation and reduce its perceived interpersonal cost. In parallel, a strong institutional tradition of team‐based practice, adherence to protocol, and a high baseline of nursing education can function as facilitators of standardized practice, provided the standards are formally endorsed and embedded into hospital‐level policy. Without such endorsement, the standards risk being treated as advisory guidance rather than performance expectations, resulting in voluntary and uneven adoption. The 9 standards, 16 criteria, and 55 indicators developed here are designed to be embedded directly into RRT‐nurse job descriptions, orientation curricula, and performance‐review instruments at the institutional level (see Section 5.1).

At the regulatory level, the most consequential constraint on the advanced nursing practice domain is the absence of independent prescriptive authority for nurses in Korea. Because domestic RRT nurses cannot independently initiate medications or diagnostic tests, time‐sensitive interventions for deteriorating patients depend on physician orders, standing protocols, or collaborative arrangements. Within this model, the scope of nurse‐led response is structurally bounded, and the corresponding indicators in the present standards are necessarily framed as activities performed under physician direction or institutional protocol rather than as autonomous acts. This regulatory boundary is the single most important factor distinguishing the operational scope of Korean nurse‐led RRTs from that of their international counterparts, where protocol‐driven prescriptive authority is more commonly available.

The recent enactment of the nursing act in Korea [34] represents a potentially significant regulatory development in this regard. While the act establishes a clearer statutory basis for the nursing profession and its scope of practice, it does not, in its current form, confer independent prescriptive authority on RRT nurses; the detailed scope of practice is to be specified through subordinate legislation and subsequent rule‐making. The proposed job standards are therefore intentionally constructed to remain compatible with the current statutory scope while also articulating practice expectations that could accommodate an expanded legal scope should future rule‐making together with the Rules on the Qualification of APNs [35] progressively broaden nurse autonomy in acute clinical decision‐making. The standards thus function as a bridging framework: applicable under present constraints, yet positioned to remain relevant if the regulatory environment evolves.

At the cross‐national level, the transferability of these standards varies with each system’s regulatory and infrastructural profile. In settings with longstanding advanced practice nursing legislation such as the United States, the United Kingdom, and Australia, comparable standards would likely be implemented through professional‐association adoption and competency credentialing, with relatively rapid uptake. In settings with more limited nursing autonomy or without formal RRT infrastructure, the standards would require additional contextual adaptation, particularly in the domains where systems differ most: prescriptive authority, activation mechanism, and qualification requirements. Accordingly, uniform global adoption is neither feasible nor desirable; the standards are better regarded as a context‐sensitive template requiring explicit organizational and regulatory adaptation prior to local implementation. Future implementation work should therefore be paired with a contextual assessment of organizational workflow, clinical decision‐making structures, and the legal scope of nursing practice in each adopting setting.

### 4.2. Core Competencies and Their Conceptual Distinctness

Nurse‐led RRTs are a relatively recent development in Korea, having first been introduced in 2008. To derive core competency standards for RRT nurses in Korea, this study integrated the Hamric model of advanced practice nursing [20] with the core competency framework for Korean APNs operationalized by the Korean Association of Advanced Practice Nurses (KABONE) [21], together with the results of an integrative evidence review of RRT nurses′ roles, activities, core competencies, and job scope. Nine core competencies were derived: advanced nursing practice, education, counseling, consultation, collaboration, leadership, evidence‐based practice, research, and ethical decision‐making.

Although these nine domains may appear to overlap, particularly advanced nursing practice and evidence‐based practice, or education, consultation, and counseling, they are conceptually and operationally distinct. Advanced nursing practice describes the direct clinical actions taken at the bedside (assessment, nursing diagnosis, nursing intervention, evaluation) for individual deteriorating patients, whereas evidence‐based practice describes the process of appraising and integrating research evidence into those clinical decisions across the RRT’s caseload. The former is performance‐oriented and case‐bound; the latter is inquiry‐oriented and trans‐case. Similarly, education refers to planned, structured instructional activities directed at patients, families, and ward staff; Consultation denotes the bidirectional exchange of expert clinical advice in response to a specific clinical question raised by or directed at another professional. Counseling is individualized, situational, supportive, and primarily informational or psychological in nature. Maintaining these as separate domains enables the standards to capture the full scope of nurse‐led RRT practice, prevents a dominant domain such as advanced nursing practice from subsuming narrower but essential domains such as research, and supports more granular performance evaluation. It should be acknowledged, however, that the boundaries between certain domains are analytic rather than absolute; in practice, activities such as evidence appraisal and bedside decision‐making frequently co‐occur, and the value of the nine‐domain structure lies in enabling differentiated assessment rather than in asserting mutually exclusive categories.

Because core competencies are widely regarded as critical determinants of professional performance, prior work has recommended that job standards be developed on the basis of the core competencies of professional nurses [36, 37]. For example, the American Nurses Association (ANA) frames its nursing standards as standards of practice and standards of professional performance, grounded in the core competencies of advanced practice nurses [18, 38]. Campo et al. [39] developed standards of practice for emergency nurse practitioners based on core competencies, scope, and ANA practice standards for advanced practice nurses in the United States. Similarly, the nursing associations in Australia and New Zealand each developed competency standards to standardize advanced practice nurses′ roles and practice, taking into account their respective national health and medical environments [37]. Additional studies have likewise developed job standards for professional nurses on the basis of core competencies [40, 41]. Taken together, these precedents indicate that competency‐based job standards can effectively reflect the distinctive characteristics of specific advanced practice nursing fields, an approach consistent with the design of the present study. At the same time, because these frameworks were developed within differing regulatory and practice contexts, their direct transferability to the Korean nurse‐led RRT setting should be interpreted with appropriate caution.

In this study, advanced nursing practice and collaboration received the highest scores in the practical‐suitability test of the draft job standards for nurse‐led RRTs. This finding is consistent with Hamric et al. [20], who identified direct clinical practice as the central core competency for advanced practice nurses, and it suggests that advanced nursing practice similarly constitutes the most fundamental competency for domestic nurse‐led RRTs. The high score for collaboration aligns with prior evidence that cooperative relationships among medical staff are associated with patient outcomes. Because nurse‐led RRTs operate within a hospital’s organizational structure, cooperation among clinical and support‐service staff is critical to their success [1]. DeVita et al. [1] argued that RRT nurses should share information on treatment plans and goals with ward staff and establish collaborative work systems to respond to deteriorating patients, and emphasized the need for nontechnical skills such as communication and leadership; RRT nurses should therefore continuously monitor and develop these skills. As this study assessed practical suitability through practitioners′ subjective ratings on a four‐point Likert scale, further research is needed to determine whether, and to what extent, the established standards are actually implemented in clinical practice.

This study indicates that RRT nurses in Korea perform activities characteristic of advanced practice nurses and require corresponding core competencies to carry out these activities effectively. At present, however, the qualifications of RRT nurses in Korea vary across hospitals. For advanced practice nurses to fulfill their intended role, they should meet consistent standards, including those related to education and experience. DeVita et al. [1] noted that educational programs should be developed and evaluated to help nurses strengthen their core competencies, thereby better preparing them to care for deteriorating ward patients. Because the core competencies required of advanced practice nurses develop gradually rather than rapidly, they are best cultivated through systematic, intensive, and planned theoretical and practical education. Establishing consistent qualification criteria and structured educational pathways may therefore support the further development of RRT nurses’ competencies, although the effect of such measures on practice and patient outcomes remains to be empirically examined.

### 4.3. Strengths, Limitations, and Methodological Reflections

Several methodological limitations should be acknowledged. First, the Delphi panel comprised eight experts drawn predominantly from academic and senior‐clinical roles. While Delphi panels of this size are within the accepted range for content validity work [26], over‐reliance on expert opinion is an inherent limitation of the method, and the perspectives of less‐experienced RRT nurses and nurses from nonacademic settings may have been underrepresented in the content‐validation step. We attempted to mitigate this through three measures: The practical‐suitability verification was conducted with 62 RRT nurses of varying experience levels including 30.6% with less than one year of RRT experience drawn from diverse hospital settings nationwide, providing a broader practitioner perspective on the standards’ applicability; the Delphi panel was deliberately composed to include both nursing and physician members of the Korean Rapid Response System Research Association, securing interprofessional input; and the job analysis step preceding the Delphi survey was conducted with currently practicing RRT nurses, ensuring that candidate items were grounded in front‐line practice rather than academic abstraction. Nonetheless, future revisions would benefit from a Delphi panel that explicitly includes early‐career RRT nurses and nurses from secondary general hospitals. Second, the field‐validation methodology relied on practitioners′ subjective ratings of suitability using a four‐point Likert scale, which captures perceived appropriateness rather than measured clinical performance; further research is required to assess whether and to what extent the standards are implemented in practice and how they relate to patient outcomes. Third, while some RRT physicians participated in the content‐validation process, they constituted a minority; future consensus‐building work including public hearings involving physicians, nurses, and representatives of the Ministry of Health and Welfare will be necessary to consolidate the standards′ authority for clinical adoption. Fourth, and most importantly for interpreting the present findings, the data underpinning these standards were collected between 2018 and 2019, and the standards were not subsequently reexamined against the current Korean RRT policy and reimbursement environment prior to submission. Since that period, the regulatory and financing context of rapid response systems in Korea including RRT‐related reimbursement provisions and the evolving legal scope of nursing practice has continued to develop, and emerging technologies such as AI‐based deterioration detection systems have become more widely adopted. Consequently, the proposed standards should be regarded as reflecting the practice context of the study period rather than the most recent regulatory state, and specific activities particularly those bearing on prescriptive authority, EMR‐based screening, and reimbursement‐linked tasks may require updating. This limitation underscores the need for a scheduled, periodic revision cycle (e.g., every 5 years or sooner in response to major policy changes) in which the standards are formally reviewed against prevailing policy and practice before continued application. Establishing such a cycle, ideally under the stewardship of an authoritative professional body, would be essential to maintaining the clinical and policy relevance of the standards over time.

## 5. Conclusions

This study was conducted to identify nurse‐led RRT nursing activities and to develop relevant job standards. These standards provide guidelines for RRT nurses’ practice levels along with a basis for task evaluation and may thereby support efforts to improve the quality of nursing care for deteriorating ward patients. This methodological study identified RRT nurses’ activities through the following procedures: an integrative evidence review, benchmarking against domestic and international institutions, and personal interviews with nurse leaders in domestic RRTs. Draft job standards were developed based on the identified activities, content validity was verified using the Delphi method (mean I‐CVI = 0.96; S‐CVI/Ave = 0.94 in Round 2), and 62 nurses from nurse‐led RRTs validated practical compatibility.

In all, 55 activities for nurses in nurse‐led RRTs were identified in nine areas, corresponding to the following nine core competencies: advanced nursing practice, education, counseling, consultation, collaboration, ethical decision‐making, research, evidence‐based practice, and leadership. The draft job standards for RRT nurses ultimately comprised 9 standards, 16 criteria, and 55 indicators after two rounds of the Delphi process. The practical‐suitability verification showed that all items in the draft job standards were more than 90% suitable. In the end, 9 job standards, 16 criteria, and 55 indicators were developed for nurse‐led RRTs.

### 5.1. Strategy for Implementation in Clinical Practice

Developing job standards is only the first step; consistent clinical adoption requires a deliberate implementation strategy. Based on the findings of this study and the experience of standard‐rollout initiatives in other nursing specialties, we propose a phased implementation roadmap comprising around four sequential phases.

First, the standards should be reviewed and formally endorsed by relevant professional bodies such as KABONE [21], the Korean Society of Critical Care Medicine, and the Korean Rapid Response System Research Association and disseminated through professional journals, conferences, and hospital nursing‐department channels. Endorsement by an authoritative professional body would help elevate the standards from advisory guidance toward a recognized professional norm, although the extent of uptake would ultimately depend on institutional willingness and regulatory alignment.

Second, hospitals operating an RRT could be encouraged through Ministry of Health and Welfare guidance and through the RRT medical insurance fee policy [14, 15] to incorporate the standards into RRT‐nurse job descriptions, orientation programs, and annual performance reviews; institutional adoption should be supported by template policy documents derived from the standards, so that hospitals do not bear the full burden of operationalization individually.

Third, the standards could inform the curriculum of RRT‐nurse training programs at both the institutional level (in‐hospital orientation) and the national level (accredited specialty courses), with competency‐based assessments aligned to the 16 criteria and 55 indicators, contributing to a coherent credentialing pipeline.

Fourth, each adopting hospital should track adherence to the standards alongside patient‐outcome indicators such as cardiac arrest rate, unplanned ICU admissions, failure‐to‐rescue, and in‐hospital mortality and report these data to a national RRT registry; such data collection would allow future studies to examine whether adherence to the standards is associated with these outcomes. Anticipated barriers include heterogeneity in hospital infrastructure, variability in nurse qualifications, hierarchical clinical cultures, and the present absence of nurse prescriptive authority. These barriers can be addressed by tiered adoption, with tertiary hospitals as early adopters and general hospitals following with adapted versions; by mentorship programs between experienced and less‐experienced RRT nurses; and by policy advocacy for expanded legal authority for RRT nurses in specified clinical scenarios. Together, the four‐phase roadmap and the corresponding mitigation strategies provide a concrete pathway from a content‐validated document to a clinically embedded standard of practice.

### 5.2. Future Research

These job standards can be used as guidelines for nurses who will work as RRT nurses in the future, as the basis for establishing desired performance levels in nursing, and as a means of evaluating the quality of nursing in nurse‐led RRTs. The standards can also inform the development of a curriculum for RRT nurses. Because this study developed and content‐validated the standards but did not test their effect on patient outcomes, three lines of follow‐up research are warranted. First, comparative effectiveness studies are needed to examine whether the deployment of nurse‐led RRTs guided by these standards is associated with changes in patient satisfaction and outcomes including mortality, cardiac arrests, unplanned ICU admissions, and failure‐to‐rescue, before and after deployment of nurse‐led RRTs, particularly in hospitals that have implemented RRTs recently. Second, as the present work is methodological, a follow‐up study is needed that uses the standards to evaluate nurses′ performance, along with a tool‐development study to evaluate the standards themselves and a multi‐center implementation study to evaluate adherence and outcomes. Third, future revisions of the standards should incorporate Delphi panels that explicitly include early‐career RRT nurses and nurses from nonacademic settings, as well as cross‐cultural studies that examine the adaptation of the standards to healthcare systems with different hierarchical, regulatory, and infrastructural characteristics.

### 5.3. Implications for Nursing Management

By identifying the nursing activities of nurse‐led RRTs and translating them into content‐validated job standards, this study offers a structured reference for defining desirable performance levels in the care of deteriorating ward patients. For nursing management, the standards provide practical guidance for both new and experienced RRT nurses and a common basis for job descriptions, orientation, workforce planning, and performance evaluation across diverse hospital settings. Because RRT nurses currently practice under inconsistent titles such as clinical nurse specialist or RRT nurse depending on institution and competency, the competency‐grounded standards can also inform more consistent qualification criteria for this role. As the standards delineate the core competencies required to manage high‐risk patients, they may further serve as a foundation for competency‐based education and for future research on nursing‐sensitive outcomes in nurse‐led RRTs. Given that evidence on the performance and cost‐effectiveness of advanced practice nurses in Korea remains limited, such work would help clarify the professional contribution of RRT nurses and, in turn, strengthen the recognized value of specialized nursing roles within the hospital workforce.

## Author Contributions

Conception and design: Eui Geum Oh and Ju‐Ry Lee; acquisition of data: Ju‐Ry Lee; analysis and interpretation of data: Eui Geum Oh and Ju‐Ry Lee; drafting the manuscript: Ju‐Ry Lee; critical revisions for important intellectual content: Eui Geum Oh, Ju Hee Lee, Yeon Soo Jang, and Yeon Hee Kim.

## Funding

The authors received no specific funding for this study.

## Disclosure

All authors approved the final approval of the version to be published.

## Conflicts of Interest

The authors declare no conflicts of interest.

## Supporting Information

Additional supporting information can be found online in the Supporting Information section.

## Supporting information


**Supporting Information** Supporting Table 1. Integrated Competency Framework for Nurse‐led Rapid Response Teams: Conceptual Definitions, Operational Scope, and Mapping to Hamric’s Model and the Korean Advanced Practice Nurse Competency Domains. Supporting Table 2. Search terms for integrative evidence review identifying nursing activities of nurse‐led RRTs. Supporting Figure 1. PRISMA flow chart of the literature‐search results.

## Data Availability

This paper is an abridged version of the first author Ju‐Ry Lee’s doctoral dissertation at Yonsei University [17]. The data used in this study are derived from the original dissertation. The data that support the findings of this study are available upon reasonable request from the corresponding author. However, the data are not publicly available due to privacy and ethical restrictions.
