# Supplementary material for: Development of Job Standards for Nurse‐Led Rapid Response Teams in the Republic of Korea
Source: J Nurs Manag. 2026 Sep 15;2026:8558376. doi: 10.1155/jonm/8558376 (PMC13575639; doi:10.1155/jonm/8558376)
Supplement: Supplementary file 1 [file JONM-2026-8558376-s001.zip › Supplementary_Table_1.docx]

Supplementary Table 1. Integrated Competency Framework for Nurse-led Rapid Response Teams: Conceptual Definitions, Operational Scope, and Mapping to Hamric's Model and the Korean Advanced Practice Nurse Competency Domains

| Domain | Conceptual Definition | Operational Definition | Mapping to Hamric's APN Model(Tracy & O'Grady, 2019 | Mapping to Korean APN Competency Framework |
| --- | --- | --- | --- | --- |
| I. Advanced Nursing Practice | The central, action-oriented competency of the advanced practice nurse, defined as the direct clinical care provided to individual patients through expert clinical judgment, comprehensive assessment, and the application of evidence-based interventions across the full cycle of clinical reasoning. | In nurse-led RRT practice, this competency is enacted at the bedside of an individual deteriorating ward patient and spans the full cycle of clinical reasoning and intervention screening and early detection, history-taking, physical examination, continuous physiological monitoring, differential clinical assessment, care planning, and the execution and evaluation of evidence-based interventions tailored to the patient's condition. Within the current legal scope of nursing practice in Korea, the RRT nurse performs assessment, clinical reasoning, and non-prescriptive interventions such as screening, proactive rounding, physical examination, and continuous monitoring autonomously, whereas prescription-dependent actions, such as initiating medications or laboratory tests, are carried out under physician orders, established protocols, or collaborative arrangements.. | **Direct Clinical Practice** | Advanced Direct Nursing Practice |
| II. Education | The competency through which the advanced practice nurse facilitates patient, family, and staff learning by guiding and coaching them through health-related transitions and the acquisition of knowledge, skills, and attitudes necessary for health management. | The competency through which the RRT nurse facilitates the acquisition of knowledge, skills, and attitudes related to the recognition and management of deteriorating ward patients among patients, families, and nursing staff, including both the act of teaching and the systematic design and evaluation of educational interventions. | **Guidance and Coaching** | Education |
| III. Counseling | The relational competency through which the advanced practice nurse provides individualized informational, emotional, and decisional support to patients, families, and colleagues facing health-related uncertainty, characterized by a dialogic and supportive interpersonal process. | The relational competency through which the RRT nurse provides information, emotional support, and decision-making assistance to patients, families, and ward nurses experiencing the uncertainty and stress associated with clinical deterioration; distinguished from education by its individualized, dialogic, and supportive character. | Guidance and Coaching | Counseling |
| IV. Consultation | The competency through which the advanced practice nurse, as a clinical expert, engages in the bidirectional exchange of professional advice — providing expertise to other healthcare providers and seeking specialist input when patient needs exceed the nurse's scope of practice. | The competency through which the RRT nurse, as a clinical expert in the management of deteriorating patients, provides professional advice to other healthcare providers (ward physicians and nurses) and, conversely, seeks expert input from other specialists when patient needs exceed the RRT's scope; bidirectional in nature, encompassing both consultant-providing and consultee-seeking roles. | Consultation | Consultation |
| V. Ethical Decision-Making | The competency through which the advanced practice nurse safeguards the ethical principles of autonomy, beneficence, non-maleficence, and justice in clinical practice, particularly under conditions of moral uncertainty or value conflict, and ensures that patients and families receive the information necessary for informed participation in care decisions. | The competency through which the RRT nurse safeguards patient autonomy, beneficence, non-maleficence, and justice in the context of acute clinical deterioration, where decisions are time-pressured and outcomes are uncertain; anchored in the patient's and family's right to accurate, evidence-based information about the patient's condition, examinations, procedures, and treatment plans as the basis for informed participation in care decisions. | Ethical Decision-Making | Ethical Decision-Making |
| VI. Research | The competency through which the advanced practice nurse contributes to the generation, dissemination, and translation of new nursing knowledge through independent and collaborative investigative activity, and through the scholarly communication of findings. | The competency through which the RRT nurse contributes to the generation, dissemination, and translation of new nursing knowledge relevant to the care of deteriorating ward patients, encompassing both independent investigative activity and collaborative participation in interdisciplinary inquiry, as well as the public dissemination of findings through conference presentations and peer-reviewed publications. | Evidence based and Research | Research |
| VII. Collaboration | The competency through which the advanced practice nurse establishes and maintains coordinated, goal-aligned, and continuous working relationships with other members of the healthcare team, characterized by shared communication systems, role clarity, and joint accountability for patient outcomes. | The competency through which the RRT nurse establishes and sustains coordinated, communicative, and goal-aligned working relationships with ward medical staff and other members of the healthcare team; distinguished from consultation by its structural and continuous character, focused on building shared systems of communication, role clarity, and joint accountability for the care of deteriorating ward patients. | Collaboration | Consultation and Collaboration: collaboration component |
| VIII. Evidence-Based Practice | The inquiry-oriented meta-competency through which the advanced practice nurse integrates the best available external research evidence with clinical expertise and patient preferences to inform clinical decisions, and systematically evaluates the outcomes of evidence application across practice over time. | The inquiry-oriented, epistemic meta-competency through which the RRT nurse appraises, integrates, and applies the best available external research evidence — together with clinical expertise and patient preferences — to inform clinical decisions in the care of deteriorating ward patients; operating as a trans-case method competency that governs how clinical decisions are generated, standardized, and audited across the RRT's caseload over time, in contrast to Advanced Nursing Practice which operates at the level of the individual patient encounter. | Evidence based and Research | none |
| IX. Leadership | The system-level competency through which the advanced practice nurse exercises influence beyond the individual patient encounter to advance clinical quality, organizational effectiveness, professional development, and health policy, encompassing clinical, professional, and organizational/policy dimensions of leadership. | The system-level competency through which the RRT nurse exercises influence beyond the individual patient encounter to shape clinical quality, organizational processes, professional development, and patient-safety policy in the context of nurse-led RRT practice, encompassing (a) clinical leadership(quality improvement, indicator management, failure-to-rescue review), (b) professional leadership(mentorship, continuing competency development, professional group participation), and (c) organizational and policy leadership(resource coordination, policy participation, guideline development). | Leadership | Leadership |

Note.RRT = rapid response team; APN = Advanced Practice Nurse; APN Model = Advanced Practice Nursing Model. The nine core competency domains were derived through the integration of Hamric's model of advanced practice nursing [reference] with the core competency domains for Korean APNs established under the Medical Service Act and operationalized by the Korean Nurses Association [reference]. Conceptual definitionsreflect the theoretical meaning of each competency as articulated in the two reference frameworks. Operational definitionsspecify how each conceptual competency was contextualized for nurse-led RRT practice in the present study, thereby serving as the a prioriclassification basis applied during the development of the job activity standards (see Section 2.3 for the study procedure). The domain labeled "Advanced Nursing Practice" corresponds to Hamric's central competency of Direct Clinical Practiceand to the Korean APN competency standard of Advanced Direct Nursing Practice); the term is used in this study to designate a specific sub-domain rather than the overarching model. The Korean APN framework subsumes both knowledge-generation and knowledge-utilization under a single "Research" domain; the present framework retains them as distinct competencies (VI. Research and VIII. Evidence-Based Practice) consistent with Hamric's later editions.
