# Supplementary material for: Development of Job Standards for Nurse‐Led Rapid Response Teams in the Republic of Korea
Source: J Nurs Manag. 2026 Sep 15;2026:8558376. doi: 10.1155/jonm/8558376 (PMC13575639; doi:10.1155/jonm/8558376)
Supplement: Supplementary file 1 [file JONM-2026-8558376-s001.zip › Supplementary_Table_2.docx]

Supplementary Table 2. Search terms for integrative evidence review to identification of nursing activities of nurse-led RRTs.

| Categories | PubMed | CINAHL | Embace |
| --- | --- | --- | --- |
| Rapid  response  team | "Hospital RRT" [MeSH]  "Medical  emergency team"  [tiab] OR “rapid response system"  [tiab] OR "rapid  response team"[tiab]  OR "critical care outreach"[tiab] | AB "Medical emergency team" OR AB “rapid response system" OR AB “RRT" OR AB "critical care outreach" | 'point of care system'/exp OR 'RRT'/exp OR 'outreach':ab,ti OR  'medical emerge-  ncy team':ab,ti |
| Nurse-led | Nurse Practitioners  [MeSH] OR “nurse specialist”[tiab]  OR Nurse[MeSH] OR nurs*[tiab] OR “nurse led”[tiab] | AB “Nurse prac-  titioner” OR AB  "nurse specialist"  OR TX “nurse  led” | 'nurse practition-  er'/exp/mj OR  'nurse practitio-  ner':ti,ab,kw OR 'clinical nurse specialist'/exp OR 'nurse specialist'  :ti,ab,kw |
